# Heterozygous and Homozygous JAK2^V617F^ States Modeled by Induced Pluripotent Stem Cells from Myeloproliferative Neoplasm Patients

**DOI:** 10.1371/journal.pone.0074257

**Published:** 2013-09-16

**Authors:** Joseph Saliba, Sofiane Hamidi, Gaëlle Lenglet, Thierry Langlois, Jingkui Yin, Xénia Cabagnols, Lise Secardin, Céline Legrand, Anne Galy, Paule Opolon, Baya Benyahia, Eric Solary, Olivier A. Bernard, Longyun Chen, Najet Debili, Hana Raslova, Françoise Norol, William Vainchenker, Isabelle Plo, Antonio Di Stefano

**Affiliations:** 1 Institut National de la Santé et de la Recherche Médicale, Unité Mixte de Recherche 1009, Laboratory of Excellence, Globule rouge-Excellence (GR-Ex), Villejuif, France; 2 University Paris-Sud 11, Le Kremlin-Bicêtre, France; 3 Institut Gustave Roussy, Villejuif, France; 4 Institut National de la Santé et de la Recherche Médicale, Unité Mixte de Recherche 951, University of Évry Val d’Essonne, Genethon, Évry, France; 5 Institut Gustave Roussy, Pathology platform, Villejuif, France; 6 AP-HP, Groupe Hospitalier Pitié-Salpêtrière, Unité Fonctionnelle de Génétique Chromosomique, Département de Génétique, Paris, France; 7 Institut National de la Santé et de la Recherche Médicale, Unité Mixte de Recherche 985, Villejuif, France; 8 Beijing Genomic Institute (BGI), Shenzhen, Shenzhen, China; Università degli Studi di Firenze, Italy

## Abstract

JAK2^V617F^ is the predominant mutation in myeloproliferative neoplasms (MPN). Modeling MPN in a human context might be helpful for the screening of molecules targeting JAK2 and its intracellular signaling. We describe here the derivation of induced pluripotent stem (iPS) cell lines from 2 polycythemia vera patients carrying a heterozygous and a homozygous mutated JAK2^V617F^, respectively. In the patient with homozygous JAK2^V617F^, additional *ASXL1* mutation and chromosome 20 allowed partial delineation of the clonal architecture and assignation of the cellular origin of the derived iPS cell lines. The marked difference in the response to erythropoietin (EPO) between homozygous and heterozygous cell lines correlated with the constitutive activation level of signaling pathways. Strikingly, heterozygous iPS cells showed thrombopoietin (TPO)-independent formation of megakaryocytic colonies, but not EPO-independent erythroid colony formation. JAK2, PI3K and HSP90 inhibitors were able to block spontaneous and EPO-induced growth of erythroid colonies from GPA^+^CD41^+^ cells derived from iPS cells. Altogether, this study brings the proof of concept that iPS can be used for studying MPN pathogenesis, clonal architecture, and drug efficacy.

## Introduction

An important breakthrough in the understanding of BCR-ABL–negative MPN has been accomplished by the discovery of the *JAK2* ^V617F^ mutation, underlying the role of pathologic JAK/STAT signaling in MPN [1,2]. The *JAK2* ^V617F^ mutation is observed in approximately 95% cases of polycythemia vera (PV) and 50-60% of cases of essential thrombocythemia (ET) or primary myelofibrosis (PMF). Most patients with JAK2^V617F^ ET harbor only one mutated allele (heterozygous), whereas most of those with JAK2^V617F^ PV harbor two mutated alleles (homozygous), supporting the hypothesis that phenotypic heterogeneity of JAK2^V617F^-induced diseases might be due to the level of JAK2 signaling [3,4]. Other recurrent mutations have also been found in genes involved in epigenetic regulation and/or in RNA splicing [5,6]. These mutations, which can be either pre-JAK2^V617F^ or post-JAK2^V617F^ events, may be involved in clonal dominance, as for *TET2* and *DNMT3A* mutations, or in disease progression as is the case for *ASXL1*, *SRSF2* or *IDH1* mutations [5,6].

Induced pluripotent stem cells (iPS) have been used to model hereditary disorders with germline mutations [7]. More recently, iPS were successfully generated from acquired malignant disorders such as chronic myeloid leukemia (CML) and non-CML MPN [8,9].

In the present study, we have generated iPS cell lines from CD34^+^ cells isolated from the blood of two MPN patients, one carrying a heterozygous and the other a homozygous JAK2^V617F^ mutation. We demonstrate that iPS cell lines are useful tools to study the clonal hierarchy, the impact of JAK2^V617F^ burden on cytokine signaling and response to small molecules.

## Results

### Derivation of human iPS cell lines from CD34^+^ cells of MPN patients and a healthy donor

Patient 1 [P1(H)] exhibited homozygous *JAK2* ^V617F^ mutation (JAK2^V617F/V617F^) in 99% of CD34^+^ cells, and presented a 20q deletion in about 25% of these cells. Using whole-exome sequence analysis, we identified an additional heterozygous *ASXL1* frameshift mutation (c.1870-1871insT:p.V624 fsX49) in 84% of CD34^+^ cells. Around 60% of CD34^+^ cells from patient 2 [P2(h)] exhibited a heterozygous JAK2^V617F^ mutation (JAK2^V617F/WT^) whereas no mutation was identified in these cells in *ASXL1* and the other genes involved in myeloid malignancies, including *TET2, EZH2, DNMT3A, IDH1* and *SRSF2* [6].

Following the protocol of Yamanaka [10], we generated iPS from these 2 MPN patients and from one healthy donor as a control. In the three cases, ES-like colonies developed that were expanded individually. Two cell lines could be obtained from patient 1, which were JAK2^V617F/V617F^ by Taqman discrimination assay. More than ten JAK2^V617F/WT^ cell lines were obtained from patient 2 (Figure S1A), of which two were selected for further analysis. We also selected 2 iPS cell lines generated from the control. The two JAK2^V617F/WT^ and the two control iPS cell lines showed a normal karyotype (Figure S1B). One JAK2^V617F/V617F^ iPS cell line (iPSa) showed a normal karyotype whereas the second (iPSb) presented an additional abnormal chromosome 20 observed in 30% of cells by FISH (Figures S1B and S1C). Accordingly, CGH array showed a normal chromosome 20 signal in iPSa cell line and a 20p^+^ in iPSb (Figure S1D). CGH array did not identify other significant differences in the iPS cell lines compared to the starting cells, in both patients and in the control (Figure S1D).

**Figure 1:**
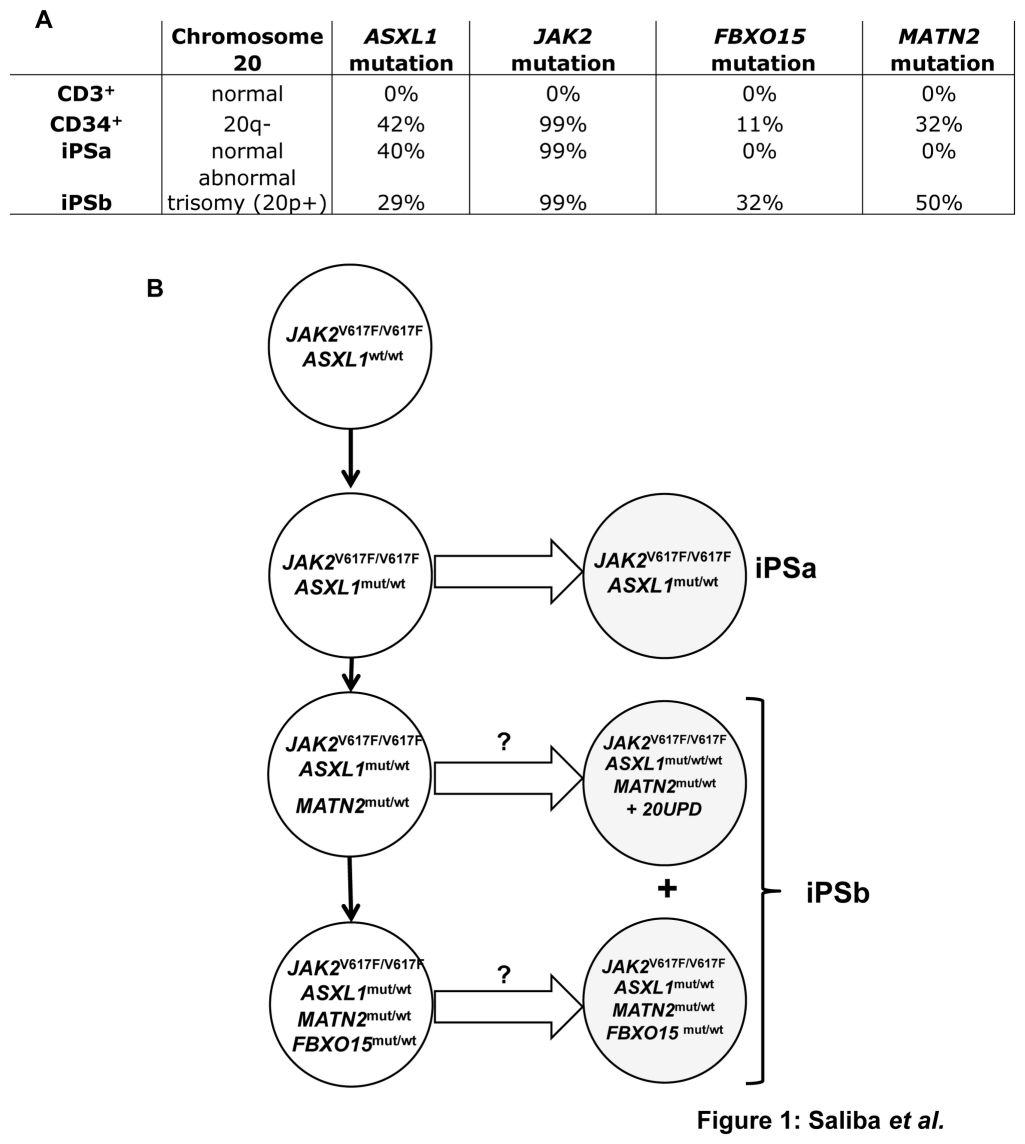
Clonal architecture of patient 1 CD34^+^ cells and origin of the iPS cell lines. (A) The allele burdens (by NGS) of the mutations and the status of chromosome 20 are indicated in CD3^+^, CD34^+^, iPSa and iPSb cell lines. (B) The *JAK2* ^V617F/V617F^ *ASXL1* ^*mut/wt*^ subclone subsequently acquired new genetic abnormalities (*FBX015* and *MATN2*) mutations and an additional abnormal chromosome 20. During reprogramming or cell culture, 2 cell lines were generated: iPSa subclone *JAK2* ^V617F/V617F^ *ASXL1* ^*mut/wt*^ and iPSb subclone *JAK2* ^V617F/V617F^ *ASXL1* ^*mut/wt*^ *FBX015* ^*mut/wt*^ *MATN2* ^*mut/wt*^ and *JAK2* ^V617F/V617F^ *ASXL1* ^*mut/wt/wt*^ *MATN2* ^*mut/wt*^ with an additional abnormal chromosome 20.

Primary and iPS cells from patients 1 and 2 were also analyzed by exome sequencing. Analysis in CD34^+^ cells compared with CD3^+^ cells showed 11 acquired mutations (*JAK2*, *LRP2*, *FBXO15*, *ZNF676*, *WNK2*, *P2RX3*, *ASXL1*, *NEURL2*, *GLYR1*, *MBLAC1* and *MATN2*) for patient 1 and 7 acquired mutations (*JAK2*, *EP400*, *CILP2*, *PPP1R1B, FHAD1*, *MICB*, *CAPN12*) for patient 2 (Tables S1 and S2). Both iPSa and iPSb cell lines from patient 2 came from the same origin cell as they bear all the mutations found in CD34^+^ cells. Patient 1 was further examined since all the mutations except those in *NEURL2* and *MBLAC1* were also found using NGS (Table S1). Both iPSa and iPSb cell lines had *ASXL1* mutations, but the mutant frequency was decreased in iPSb compared to iPSa (29% versus 40%, respectively) due to the additional gene copy of *ASXL1* in 1/3 of the cells (Figure 1A). Both iPSa and iPSb developed from a *JAK2* ^V617F/V617F^ *ASXL1* ^mut/wt^ subclone, as attested by the presence of the same *ASXL1* mutation in the two cell lines. The iPSb cells originated from a genetically more advanced cell that had acquired two additional mutations (*FXB015* and *MATN2*) (Figure 1A). The additional abnormal chromosome 20 might have been acquired either during culture or reprogramming, or was already present in the reprogrammed CD34^+^ cell. In this last case the iPSb cell line may not be clonal and may originate from two cells as indicated by the *FBX015* mutation burden (32%) (Figure 1A). Altogether, studies of mutation burden and iPS genotype suggest a clonal hierarchy in the CD34^+^ cells from patient 1 as shown in Figure 1B.

Exome sequencing of the 4 iPS cell lines derived from MPN patients identified an average of 10 mutations acquired during reprogramming, as they were not detected in the primary CD34^+^ cells. This analysis confirmed that all iPSa and iPSb cell lines were independently generated, as they did not bear the same acquired mutations. A similar number of acquired mutations during reprogramming was identified in the control iPS indicating all the iPS cell lines were genetically relatively stable.

**Figure 2:**
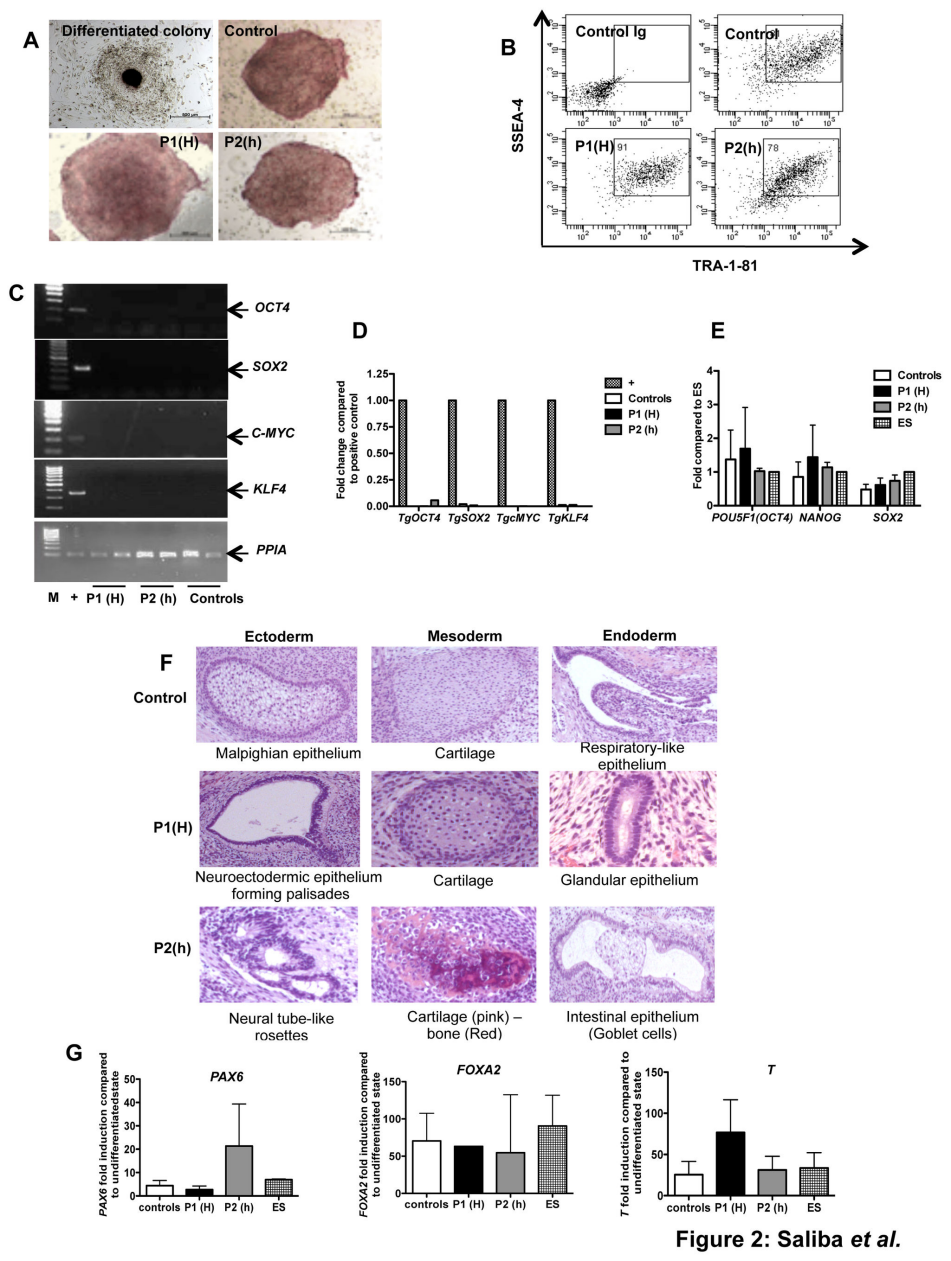
Biological characterization of iPS cell lines (A) Analysis of AP activity in iPS colonies from P1(H), P2(h) or control *versus* a differentiated colony. (B) Flow cytometry analysis of TRA-1-81 and SSEA-4 pluripotency markers. (C) RT-PCR performed on expression of the exogenous transgenes Tg*SOX2*, Tg*OCT4*, Tg*KLF4* and Tg*C*-*MYC* in undifferentiated iPS cells using specific primers. Positive controls correspond to 293EBNA cells transduced with the retroviral vectors. (D) Fold changes of exogenous transgenes (Tg*SOX2*, Tg*OCT4*, Tg*KLF4* and TgC-*MYC*) were compared to positive controls in iPS-derived GPA^+^ cells by qRT-PCR. (E) Endogenous pluripotent transcription factors (*NANOG*, *POU5F1* and *SOX2*) fold changes in undifferentiated iPS were compared to ES cells by qRT-PCR. (F) Teratomas were analyzed after hematoxylin*-*eosin*-*saffron staining. (G) Analysis of spontaneous differentiation of iPS and ES cells by *BRACHYURY* (*T*), *FOXA2* and *PAX6* gene expression at day 5. Results are expressed as fold changes compared to undifferentiated state (mean ± SEM, n=4).

We then investigated the pluripotency of the 4 MPN-derived and the 2 control undifferentiated iPS cell lines, which behave similarly in culture (Figure 2). All these cell lines expressed high levels of alkaline phosphatase (AP) (Figure 2A) and cell surface pluripotency markers including TRA-1-81 and SSEA-4 (Figure 2B). RT-PCR and qPCR analyses showed the silencing of the four reprogramming transgenes in all iPS cell lines, not only at the undifferentiated state, but also during hematopoietic differentiation (Figure 2C and D, respectively). All iPS cell lines expressed the endogenous pluripotency-associated genes such as *POU5F1, NANOG* and *SOX2* at levels comparable to ES cells (Figure 2E). In addition, expression of GFP from the viral vectors was undetectable. Pluripotency was further strengthened by biological assays: *in vivo* teratoma formation with the generation of the three germ layer tissues (Figure 2F); *in vitro* generation of embryoid bodies expressing the three germ layer markers by qPCR at levels identical to those observed with ES cells (Figure 2G).

Altogether, these results demonstrate that we have generated *bona fide* JAK2^V617F/WT^ and JAK2^V617F/V617F^ iPS cell lines from MPN patients. To evaluate if they were valuable tools for studying the cytokine hypersensitivity of MPN cells and drug screening, we analyzed their hematopoietic differentiation potential.

### Hematopoietic differentiation

**Figure 3:**
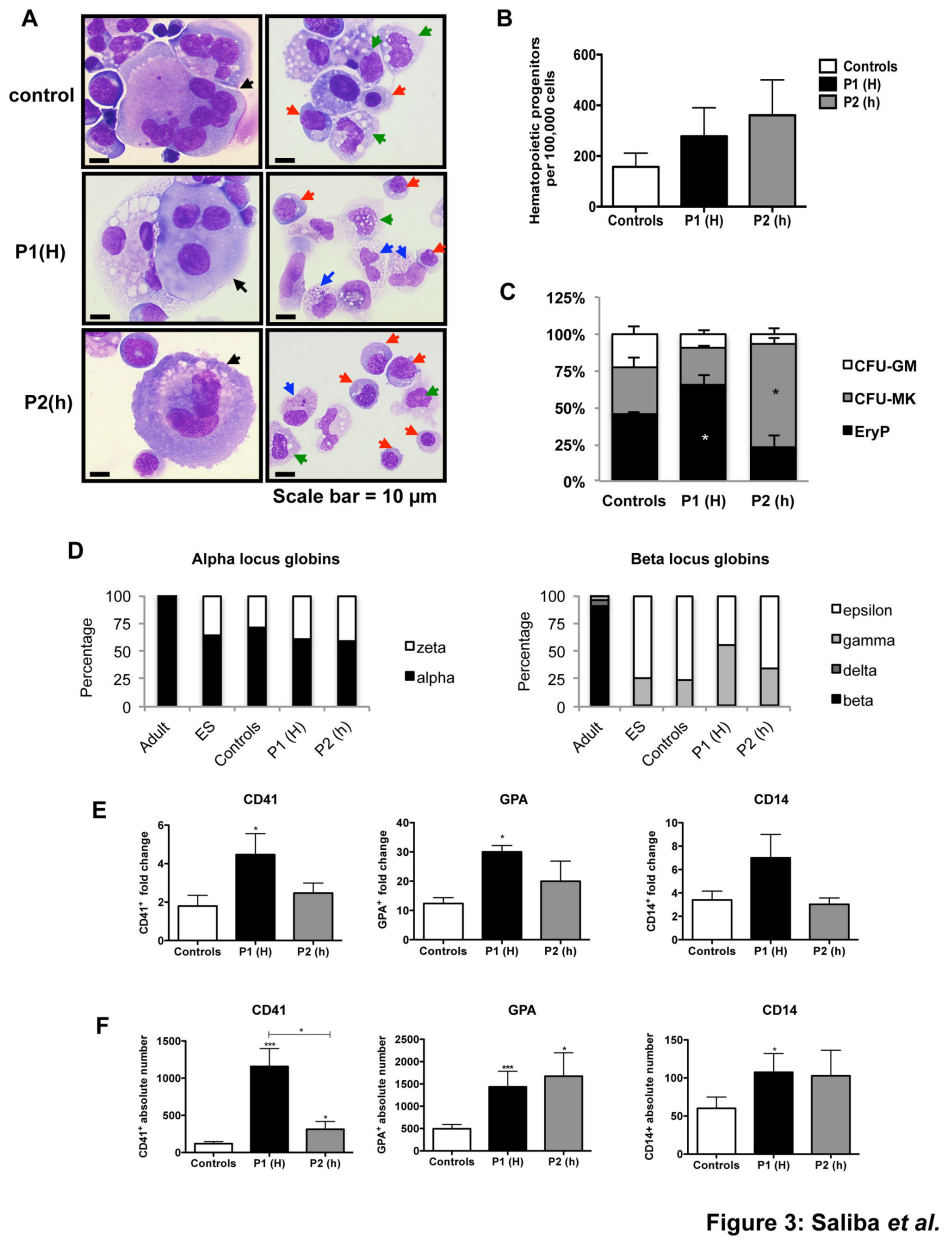
Hematopoietic differentiation of iPS cell lines (A) Cytological analysis showing EryP (red arrows), MK (black arrows), monocytes (green arrows) and granulocytes (blue arrows). (B) iPS cells from P1(H), P2(h) or control were induced for hematopoietic differentiation with EPO (1 U/mL), TPO (20 ng/mL), SCF (25 ng/mL) and IL-3 (10 ng/mL) till day 13. 1x10^5^ cells were plated in semi-solid conditions (both methylcellulose and plasma clots) and hematopoietic progenitors were counted in both conditions at day 12 and 10, respectively. (C) Proportions of each progenitor (CFU-MK, CFU-GM and EryP) was calculated for each iPS cell line. (D) Percentage of alpha and beta locus globins was calculated after qRT-PCR in iPS and ES-derived GPA^+^ cells or adult CD34^+^-derived erythroblasts. (E) Fold changes represent the amplification of CD41^+^ GPA^+^ cells at day 12 into CD41^+^ cells or GPA^+^ cells at day 18. Alternatively, fold changes represent the amplification of CD14^+^ cells between days 15 and 21. (F) Hematopoietic differentiation potential of iPS cells was quantified by plating one TRA-1-81^+^ cell per well in a 96-well plate coated with OP9 stromal cells. The absolute number of CD41^+^, GPA^+^ and CD14^+^ cells in each clone was measured by flow cytometry at day 18 (mean ± SEM, n>10; 2 independent experiments) (Mann Whitney test, two-tailed, * P<0.05, ***P<0.001).

Hematopoietic differentiation was performed on the OP9 cell line [11]. Sac-like structures were obtained, dissociated at day 12 and further cultured on OP9 cells in the presence of cytokines. Two iPS cell lines of each genotype were studied and the results were combined in each experiment. Kinetics analysis performed between day 10 and day 21 did not reveal marked differences in the expression of hematopoietic markers such as CD34, CD43, GPA, CD41, CD15 and CD14 among the different control and JAK2^V617F^ iPS cell lines. More specifically, GPA^+^CD41^+^ hematopoietic cells reached a maximal amplification at days 11-12 and progressively declined in percentage with the appearance of GPA ^+^ CD41^-^ cells (erythroblasts) and CD41^+^CD42^+^GPA^-^ cells (MK) as well as granulo-monocytic cells (CD14^+^ and CD15^+^ cells) (Figure 3A). These results were similar to those previously obtained with human ES cells [12,13]. To evaluate the presence of hematopoietic progenitors, unfractionated cells were seeded in semi-solid medium in presence of cytokines and colonies were enumerated at day 10-12. No marked differences were observed in the number and the size of colonies between the different cell lines (Figure 3B). Compared to control iPS cell lines, the progenitor distribution was shifted towards CFU-MK or erythroid progenitors for JAK2^V617F/WT^ or JAK2^V617F/V617F^ iPS, respectively (Figure 3C). Erythroid colonies (EryP) were typically small as expected for their primitive hematopoiesis origin. To test this hypothesis, we evaluated by qRT-PCR the expression levels of globin transcripts (zeta, alpha, epsilon, gamma, delta and beta) in erythroblasts derived from patient 1, patient 2 and the control iPS cell lines in comparison with erythroblasts derived from ES and adult cells. The synthesis of ε and γ globin chains in the absence of β chains, as well as the synthesis of ζ chains, confirmed the embryonic pattern of globin chain expression in control and JAK2^V617F^ iPS cell lines (Figure 3D).

To evaluate the hematopoietic cell output from the different iPS cell lines, we performed two types of assays. In the first set of experiments, we calculated the amplification in the number of erythroblasts (GPA^+^) and MK (CD41^+^) generated at day 18 from their day 12 common progenitors (GPA^+^CD41^+^ cells). Because monocytes differentiate later, their amplification was analyzed between days 15 and 21 (Figure 3E). A slight increase in the amplification of erythroblasts and MKs was observed for both JAK2^V617F/V617F^ and JAK2^V617F/WT^ iPS lines, but reached statistical significance only for the JAK2^V617F/V617F^ genotype (Figure 3E). The second set of experiments was designed to more accurately quantify the hematopoietic potential of iPS cell lines. TRA-1-81^+^ iPS cells were sorted and cloned on OP9 stromal cells with a cocktail of cytokines. Each clone was recovered after 18 days and the absolute number of different cells was determined by flow cytometry. The generation of the GPA^+^ and CD41^+^ cells was markedly and significantly increased in JAK2^V617F/V617F^ and JAK2^V617F/WT^ as compared to the control cell line. In addition, the increase in CD41^+^ cells and CD14^+^ cells was stronger in the JAK2^V617F/V617F^ cell lines (Figure 3F). Together, our data show that JAK2^V617F^ mutation induces a moderate, but significant increase in the output of the three myeloid lineages, especially when homozygous.

### JAK2^V617F^ increases sensitivity to EPO and TPO

MPN are characterized by independency or hypersensitivity of hematopoietic progenitors to several cytokines, especially EPO and TPO [14]. EEC (endogenous erythroid colonies) have been used for several years as an assay for PV diagnosis [14]. To check if MPN-derived iPS cell lines would reproduce the properties of the primary cells, erythroid and MK progenitors derived from iPS were evaluated for their response to EPO and TPO.

**Figure 4:**
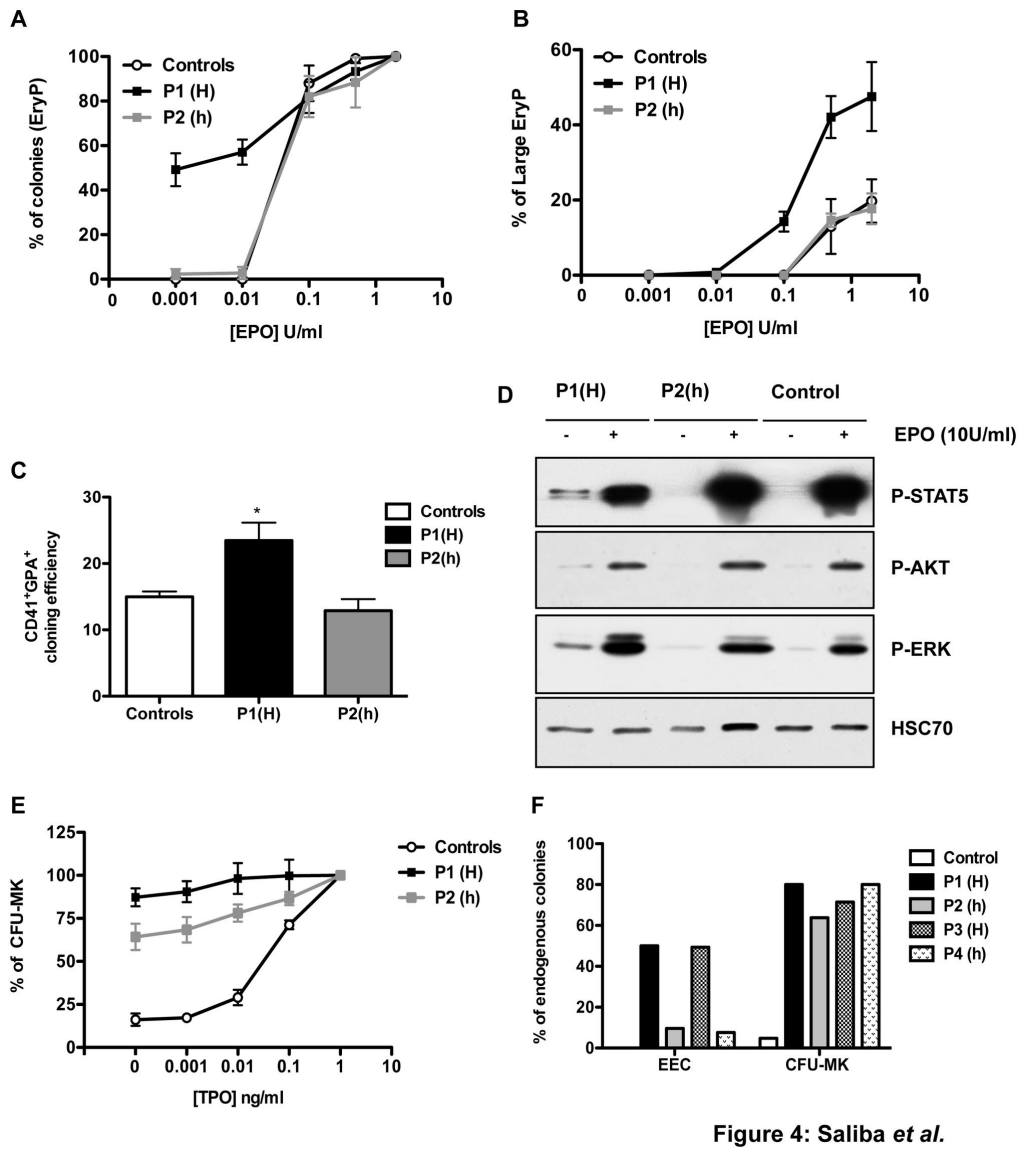
JAK2^V617F^ induces an increased sensitivity to EPO and TPO (A) GPA^+^CD41^+^ cells from P1(H), P2(h) or control were plated in methylcellulose in the presence of SCF (25 ng/mL) and increasing concentrations of EPO. EryP colonies were counted 12 days later. (B) The percentage of large EryP (>50 cells per colony) in total EryP was also calculated. Results are the mean ± SEM of 3 independent experiments. (C) Cloning efficiency of GPA^+^CD41^+^ cells for each genotype was calculated (D) GPA^+^ cells were cytokine-deprived overnight in serum-free medium and then seeded in IMDM alone for 4 hours. Cells were then stimulated with 10 U/mL EPO or not. (E) CD41^+^ cells from P1(H), P2(h) or control were plated in plasma clots without or with increasing concentrations of TPO. CFU-MK colonies were counted at day 10 after CD41a indirect staining. Results are the mean ± SEM of 3 independent experiments (*P<0.05). (F) Primary cells from one control and 4 patients (P1(H), P2(h), P3(H) and P4(h)) were grown either with SCF (25 ng/mL) ±EPO (1 U/mL) or with SCF (25 ng/mL) ± TPO (20 ng/mL), and cloned at one progenitor cell/well. The percentage of endogenous erythroid colonies (EEC) or endogenous CFU-MK was calculated for each condition.

The iPS cell lines were grown on OP 9 stromal cells for 12 days in presence of VEGF and hematopoietic cytokines. At day 12, the progenitor cell fraction GPA^+^CD41^+^ was sorted and grown for 12 days in methylcellulose in the presence of SCF and increasing concentrations of EPO. EEC were only observed in the JAK2^V617F/V617F^ cell lines, associated with a hypersensitivity to EPO (Figure 4A). Moreover, the percentage of large EryP colonies was higher in JAK2^V617F/V617F^ cell lines compared to JAK2^V617F/WT^ and control iPS cell lines (Figure 4B). In contrast, no differences in EPO sensitivity could be observed between JAK2^V617F/WT^ and control iPS cell lines. Finally, JAK2^V617F/V617F^ exhibited a higher cloning efficiency when compared with JAK2^V617F/WT^ and control iPS cell lines (Figure 4C).

JAK2 is a key molecule in the cytokine-receptor signaling cascade and JAK2^V617F^ induces a constitutive activation of downstream signaling pathways in cell lines and primary cells [1]. When erythroblasts were expanded in liquid cultures in the presence of EPO and SCF from day 12 till day 18, then recovered and deprived of cytokines for 24 hours, a constitutive phosphorylation for STAT5, ERK and AKT was restricted to JAK2^V617F/V617F^ cells (Figure 4D). EPO induced the phosphorylation of these three proteins in all the erythroblasts, whatever their genotype.

To explore TPO sensitivity of MK progenitors, we used a similar protocol as for erythroid cells, but the clonogenic assays were performed in serum-free fibrin clots in the absence or presence of increasing TPO concentrations (Figure 4E). Rare TPO-independent MK colonies, around 10% of maximally TPO-stimulated cultures, were obtained from MK progenitors generated from control iPS cell lines. In contrast, 70% and 90% TPO-independent MK colonies were observed for JAK2^V617F/WT^ and JAK2^V617F/V617F^ iPS cells, respectively (Figure 4E). In these experiments, no difference was observed between iPSa and iPSb JAK2^V617F/V617F^ cells.

To confirm these results, we also studied the endogenous growth of EEC and CFU-MK from CD34^+^ progenitors not only of P1(H) and P2(h) but also of 2 other patients with only homozygous P3(H) or heterozygous P4(h) colonies. Cells from all patients were independent to TPO as examined by two other techniques either in MegaCult-C collagen (data not shown) or in serum-free liquid medium with SCF±TPO after plating one progenitor cell/well. In contrast, only the cells from homozygous patients were clearly independent to EPO (Figure 4F).

Together, our data demonstrate that JAK2^V617F^ heterozygosity profoundly modifies the response to TPO, but not to EPO.

### Effect of JAK2 and JAK2 receptor signaling inhibitors

**Figure 5:**
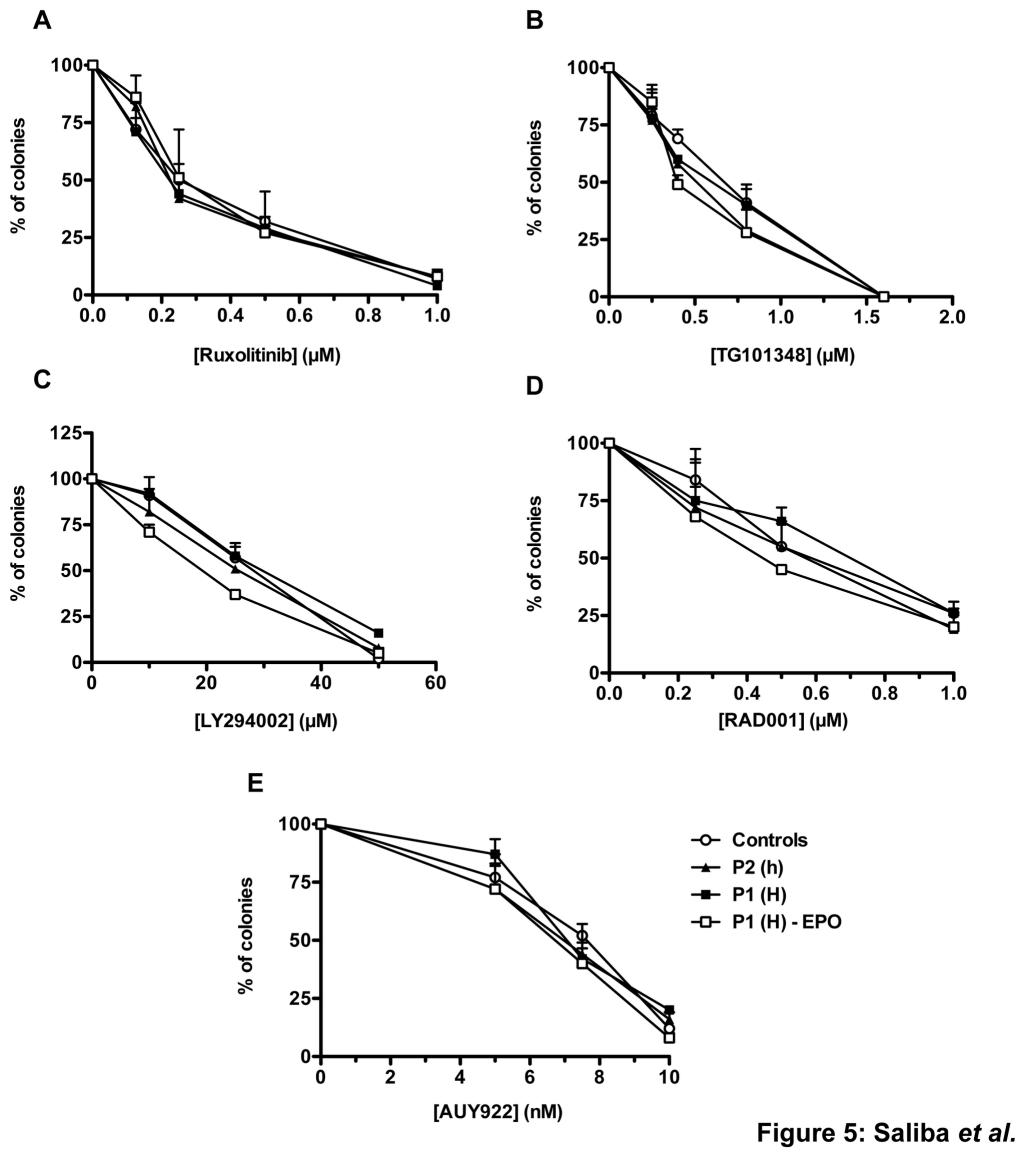
Impact of different inhibitors on erythroid growth GPA^+^CD41^+^ cells were plated in methylcellulose with various inhibitors, in the presence of SCF (25 ng/mL), without EPO for P1(H), and with EPO (1 U/mL) for P1(H), P2(h) and control. EryP colonies were counted 12 days later. (A) Ruxolitinib ; (B) TG101348 ; (C) LY294002 ; (D) RAD001 ; (E) AUY922. Results are the mean ± SEM of 3 independent experiments (*P<0.05).

Finally, we asked if JAK2^V617F^ iPS cell lines could be used for testing small molecules targeting JAK2 or its signaling. For this purpose, we tested different inhibitors targeting signaling pathways, some of them being introduced in clinical trials or practice [15,16]. These molecules were Ruxolitinib (Figure 5A), a JAK2 and JAK1 inhibitor, TG101348 (SAR302503) (Figure 5B), a JAK2 and FLT3 inhibitor, Ly294002, a pan PI3 kinase inhibitor (Figure 5C), RAD001, a mTOR inhibitor (Figure 5D) and AUY922, an HSP90 inhibitor (Figure 5E). Dose-response studies were performed on the growth of erythroid colonies from GPA^+^CD41^+^ progenitors in the presence of EPO for all iPS cell lines and also on EEC from the JAK2^V617F/V617F^ iPS cell lines. All of these 5 small molecules inhibited erythroid growth in a dose dependent manner. We did not observe any difference in the sensitivity to these inhibitors between the different cell lines, whatever their genotype.

## Discussion

The generation of human iPS cell lines has opened opportunities to model several monogenic hereditary disorders from patient fibroblasts [7]. More recently, the successful reprogramming of peripheral blood cells such as CD34^+^ cells, monocytes and T cells allowed the generation of iPS cell lines that model acquired blood disorders [17,18]. In particular, iPS cell lines derived from CML or JAK2^V617F^ MPN CD34^+^ cells have been reported [8,9]. The present study allows: i) to quantify the induction of hematopoiesis induced by iPS generated from MPN patients with either heterozygous or homozygous JAK2^V617F^ mutation, ii) to investigate how JAK2 signaling intensity generates disease heterogeneity and iii) to develop new tools to test small molecules.

To generate iPS cell lines, we ectopically expressed four transcription factors (*OCT4, SOX2, KLF4* and *c-myc*) from Moloney-derived retroviruses [10]. Although simple and efficient for CD34^+^ cells, this technique could have two main disadvantages. First, the methylation-induced silencing of the 4 transgenes that occurs during reprogramming might be incomplete and transgenes might be re-expressed during differentiation [19]. We checked that transgene silencing was almost complete in both undifferentiated and differentiated hematopoietic cells. Second, integration sites of the retroviruses may affect the hematopoietic differentiation, which explains why we studied two independent iPS cell lines derived from the same patient. In the future, the use of recently described non-integrative techniques to generate iPS cell lines may avoid these potential pitfalls [20].

The heterozygous JAK2^V617F/WT^ and homozygous JAK2^V617F/V617F^ iPS cell lines were derived from selected patients. In patient 1, whose PV had progressed to myelofibrosis, JAK2^V617F^ burden was around 100% in CD34^+^ cells and a 20q deletion was detected in a fraction of these cells. Patient 2 had a PV with an important thrombocytosis and a heterozygous JAK2^V617F^ mutation in all tested progenitor-derived colonies [3]. Since the discovery of JAK2^V617F^, several other genetic mutations have been identified in MPN making the molecular pattern more complex [5,6]. We used exome sequencing to establish the mutation landscape and clonal architecture of the proliferations. These experiments demonstrated that the two iPS cell lines (iPSa and iPSb) derived from patient 1 CD34^+^ cells originated from a dominant *JAK2* ^V617F/V617F^ *ASXL1* ^mut/wt^ clone displaying a series of common allele variants at a frequency of 50%, except two of them (*FXBO15* and *MATN2*), which were absent in the iPSa cell line. These results indicated that iPSb represents a slightly more advanced stage of the disease. In addition, iPSb cell line harbored 30% of cells with an additional abnormal chromosome 20 suggesting that they may be derived from two cells. Altogether, this analysis demonstrates that the generation of iPS cell lines may allow isolation of distinct subclones and thus to model oncogenic cooperation in human. One intrinsic limitation to this approach is that during reprogramming some somatic coding mutations may occur. The 10-20 point mutations identified in each exome were slightly higher than previously reported in iPS-derived from human and murine fibroblasts (in average 2-6), but was equivalent in all our iPS cell lines, including controls [21,22].

Interestingly, the number and repartition of *ASXL1* gene alleles differed in iPSa (*ASXL1* ^mut/wt^) and iPSb (*ASXL1* ^mut/wt/wt^) clones. Since *ASXL1* mutations lead to haploinsufficiency [23], iPSb *ASXL1* ^mut/wt/wt^ can be considered as a kind of *ASXL1* revertant. A similar approach has been used to study the role of trisomy 21 on hematopoiesis by generating isogenic iPS subclones except for the number of chromosome 21 [24,25]. Despite this variation in the *ASXL1* copy number, we did not find any significant differences in differentiation properties and the cytokine response between iPSa and iPSb cell lines, in agreement with a recent report showing the lack of effect of *ASXL1* knock-down on the erythroid and monocytic *in vitro* differentiation from human CD34^+^ cells [26].

Overall, MPN-derived iPS demonstrated an increased hematopoietic potential suggesting an enhanced response of hematopoietic progenitors to cytokines when the JAK2^V617F^ mutation is present. Here, we show that, in the JAK2^V617F/V617F^ background, the growth of erythroid and MK progenitors is almost completely independent of EPO and TPO, respectively. In the JAK2^V617F/WT^ background, this independence is limited to TPO. To confirm that these difference in EPO and TPO responses in iPS derived hematopoietic cells were not a bias induced by cellular reprogramming, we studied primary cells of the two patients and from two other patients with homozygous and heretozygous mutations and confirmed these results. These differences according to the number of JAK2^V617F^ copies might explain why heterozygous mutations are essentially associated with ET and homozygous mutations with PV [3,4]. Other determinants that could participate to the phenotype include the level of STAT1 activation [27]. In line with our finding, JAK2 germline mutations with a kinase activity lower than JAK2^V617F^ and a specific activation of STAT1 were identified in hereditary thrombocytosis [28].

iPS cell lines generated from malignant MPN progenitors are shown to retain properties that might be used to screen molecules in preclinical trials [29,30,31,32]. Dose-response studies performed on the growth of erythroid colonies with two JAK2 inhibitors, two PI3Kinase/mTOR inhibitors and one HSP90 inhibitor provided results that were in agreement with the literature [33]. The lack of major difference in the sensitivity of erythroid progenitors derived from normal or MPN iPS cell lines to these inhibitors further suggests that none of these inhibitors is specific towards JAK2^V617F^ or its signaling. These results contrast with the higher sensitivity to these inhibitors in the CD34^+^ progenitors from MPN patients compared to controls in some studies [34,35,36]. In fact the response to inhibitors are mainly dependent of the EPO concentration used. Indeed EPO independent growth is more sensitive to JAK2 inhibitors than EPO stimulated growth. However at high EPO concentration there is no difference between JAK2V617F and JAK2WT progenitors [37]. However in the present study we did not find a significant difference in the EPO independent and EPO dependent growth. These discrepancies could be due to the different types of progenitors used CD41^+^GPA^+^ primitive progenitors versus adult CD34^+^ progenitors or to the use of SCF in our assay. Alternatively, the mutations acquired during reprogramming might play a role in the sensitivity towards inhibitors. However, this second hypothesis seems unlikely because each clone has acquired different and independent mutations. A panel of JAK2^V617F^ iPS clones with different associated mutations in epigenetic or RNA splicing genes could be helpful testing new drugs and drug combinations aiming at decreasing *JAK2*-mutated MPN disease burden.

In conclusion, the present study confirmed the feasibility of iPS cell lines generation from peripheral blood CD34^+^ cells of JAK2^V617F^ patients. These iPS cell lines allowed demonstrating differences in the EPO and TPO responses between homozygous or heterozygous JAK2^V617F^ cells. This approach also allowed testing the effects of small molecules on the growth of JAK2^V617F^ progenitors.

## Materials and Methods

### Patients, iPS generation and cultures

All participants to this study gave their written informed consent in accordance with the Declaration of Helsinki and the study was approved by the «Local Research Ethics Committee from the Hôtel-Dieu Hospital» (Paris, France) and by «the Programme Hospitalier de Recherche Clinique (PHRC)». MPN was defined following WHO criteria for PV and myelofibrosis [38]. Blood from a healthy donor was used as control. CD34^+^ and CD3^+^ cells were purified from blood mononuclear cells. CD34^+^ cells were cultured in serum-free medium with cytokines for five days before being infected with VSV-G pseudotyped retroviruses encoding *Oct4*, *c-myc*, *Klf4* and *Sox2* [10]. Six days later, cells were seeded on irradiated murine embryonic fibroblasts (MEF) in ES medium [39]. Colonies with an ES-like morphology were picked from day 20 to day 30 and expanded.

Hematopoietic differentiation was performed on OP9 stromal cells in the presence of VEGF (20 ng/mL) (Peprotech, Neuilly-sur-Seine, France) [11]. On day 7, EPO (1 U/mL) (Amgen, Thousand Oaks, CA), TPO (20 ng/mL) (Kirin, Tokyo, Japan), SCF (25 ng/mL) (Biovitrum AB, Stockholm, Sweden) and IL-3 (10 ng/mL) (Miltenyi Biotec, Paris, France) were added and on day 11-12, cells were enzymatically dissociated. The recovered cells were cultured or sorted on the expression of GPA and CD41. Clonal differentiation of iPS was also performed on OP9 cells.

### Quantification of clonogenic progenitors in semi-solid cultures

Cells were plated either in methylcellulose with SCF and EPO to quantify erythroid (EryP) and granulo-monocytic (CFU-GM) progenitors or in serum-free fibrin clot assays with TPO to quantify CFU-MK [40]. Cultures were scored after 12-14 days for EryP and CFU-GM-derived colonies [12]. MK colonies were enumerated at day 10 after labeling by an indirect immuno-alkaline phosphatase staining technique using an anti-CD41a monoclonal antibody (Becton Dickinson, clone HIP8), as previously described [40] or by commercial MegaCult-C collagen (Stemcell Technologies, Grenoble, France).

### Teratoma assays and embryoid bodies

iPS cells (1x10^6^) were scrapped and resuspended in 140 µL ES medium. Undiluted matrigel (60 µL) was added prior to subcutaneous injection into Rag2^-/-^ γC^-/-^ mice. After 8-12 weeks, tumors were isolated and fixed in formalin (10%). Sections were stained for germ layers analysis. Spontaneous differentiation was generated by embryoid body (EB) formation [12].

### Antibodies and flow cytometry analysis

Directly conjugated monoclonal antibodies were used for iPS cell (SSEA4, eBioscience, San Diego, CA and TRA-1-81, Becton Dickinson, le Pont de Claix, France), for sorting and characterization of hematopoietic cells (anti-CD34, Beckman, Villepinte, France; anti-CD43, CD42 and GPA, Invitrogen, Cergy-Pontoise, France, and anti-CD41, CD15 and CD14, Pharmingen, San Diego, USA). Cells were sorted on an Influx flow cytometer (Becton Dickinson) and analyzed on a FACS Canto II (Becton Dickinson). iPS colonies were stained by an alkaline phosphatase (AP) reaction (Stemgent, Cambridge, MA, USA).

### Quantitative real-time PCR, karyotype, FISH, CGH arrays, whole-exome sequencing (WES), next generation sequencing (NGS)

Total RNA was isolated using RNeasy Mini Kit (Qiagen, Courtaboeuf, France) and cDNA was synthesized by SuperScript II Reverse Transcriptase (Invitrogen). PCRs were carried out in the ABI Prism GeneAmp 7500 Sequence Detection System (Applied Biosystem, Invitrogen), using the Power SYBR Green PCR Master Mix (Invitrogen) and specific primers (Table S3). All genes were expressed relatively to *PPIA* or *HPRT*.

Genomic DNA was isolated using DNA QiaAmp Kit (Qiagen). A TaqMan allelic discrimination assay was used for JAK2^V617F^ as previously described [1]. CGH arrays from purified CD34^+^ cells or iPS cell lines derived from patients were performed on human CGH 2x400K (G4448A) by hybridization of sample *versus* normal-matched commercial reference and a hierarchical clustering was performed (E-MTAB-1494). The CD34^+^ cells and the derived iPS cells generated in this study were done after patients gave their written informed consent in accordance with the Declaration of Helsinki and with respect to taking the samples and making the cell line. These experiments were approved by the «Local Research Ethics Committee from the Hôtel-Dieu Hospital» (Paris, France) and by «the Programme Hospitalier de Recherche Clinique (PHRC)». The WES was performed using HiSeq2000 after capture with Agilent kit (Beijing Genomic Institute (BGI), Beijing, China). We analyzed the results by comparing CD3^+^ non-tumoral cells to CD34^+^ cells or to iPS cell lines and CD34^+^ to iPS cell lines. DNA sample were then amplified with sets of primers tagged with a unique and identical bar code for each cellular fraction to generate a library. Amplicons were sequenced using ion torrent sequencing to quantify the variants (average 10 000 reads).

Karyotypes were performed using standard procedures on R-banded metaphases (450-600 bands). FISH analysis was performed using Whole Chromosome Painting probes on an Octochrome device allowing the simultaneous analysis of the whole genome in one hybridization, and when necessary, chromosome 20 was further analyzed with different specific probes.

### Western blot analysis

Signaling studies were performed on cultured erythroblasts after overnight cytokine deprivation in serum-free medium. Restimulation by EPO (10U/mL) for 15 minutes was done as positive control. Samples were subjected to Western blot analysis using polyclonal antibodies against the phosphorylated forms of STAT5 (Tyr 694), ERK1/2 (Thr 202/Tyr 204), AKT (Ser 473) (Ozyme). HSC70 was used as loading control and was from Stressgen (Victoria, Canada).

### Small molecules

Increasing concentration of small molecules inhibitors was tested in erythroid colony assays in the presence of SCF and with or without EPO. Two JAK2 inhibitors (Ruxolitinib and TG101348 renamed SAR302503), a pan PI3K inhibitor (LY294002), a mTOR inhibitor (RAD001) and a HSP90 inhibitor (AUY922) were tested. All inhibitors were from Selleck chemicals (Euromedex, Souffelweyersheim, France).

## Supporting Information

Figure S1 **Molecular characterization of iPS.** (A) JAK2^V617F^ genotyping of two iPS cells from P1(H), P2(h) or control using a real-time PCR single nucleotide polymorphism detection system. For controls, DNA from a JAK2^V617F/V617F^ mutated sample from the HEL cell line was combined in various proportions with JAK2^wt^ DNA from K562 cells. (B) Karyotypes of iPS from control, P1(H) and P2(h). (C) FISH analysis of iPSb from P1(H) hybridized with subtelomere specific probes for chromosome 20 p-arm (green) and q-arm (red) and BlueFish proximal specific probes RP11-108H13 (red) and RP11-327D19 (green). (D) CGH array analysis is represented by hierarchical Clustering with Pearson distance on 9 samples including two iPS clones and their respective CD34^+^ progenitors cells from P1(H), P2(h) and control. Dots correspond to amplifications (in green, *log2ratio>1.5*) and deletions (in red*, log*_*2*_ *r*<-1.5) and lines correspond to gains (in blue, 0<log2ratio<1.5) and loss (in red, 0>log2r>-1.5).
(DOCX)

Table S1 **Exome analysis of Patient 1 [P1(H)].** This table indicates the analysis of variants found by exome sequencing in CD3^+^, CD34^+^, iPSa and iPSb of Patient 1 [P1(H)]. The allele burdens of the variants were then validated by NGS.
(XLSX)

Table S2 **Exome analysis of Patient 2 [P2(h)].** This table indicates the analysis of variants found by exome sequencing in CD3^+^, CD34^+^, iPSa and iPSb of Patient 2 [P2(h)].
(XLSX)

Table S3 **Table of primers.** All the primers used in the study are listed.
(XLSX)
