# Supplementary figures and images for: Heterozygous and Homozygous JAK2V617F States Modeled by Induced Pluripotent Stem Cells from Myeloproliferative Neoplasm Patients
Source: PLoS One. 2013 Sep 16;8(9):e74257. doi: 10.1371/journal.pone.0074257 (PMC3774801; doi:10.1371/journal.pone.0074257)

**SUPPORTING FIGURES**


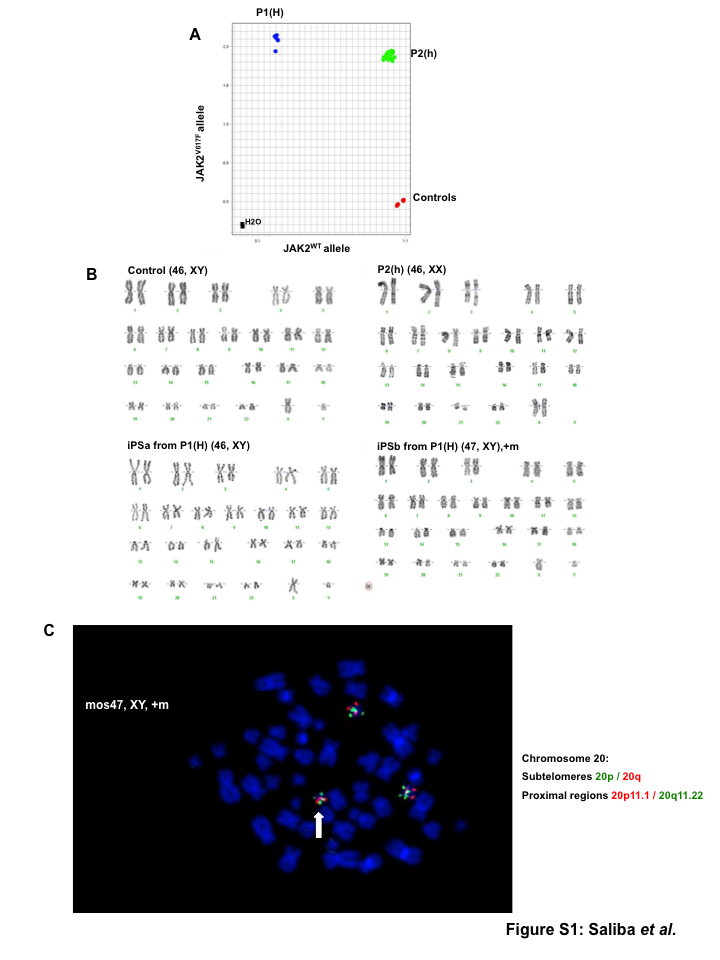


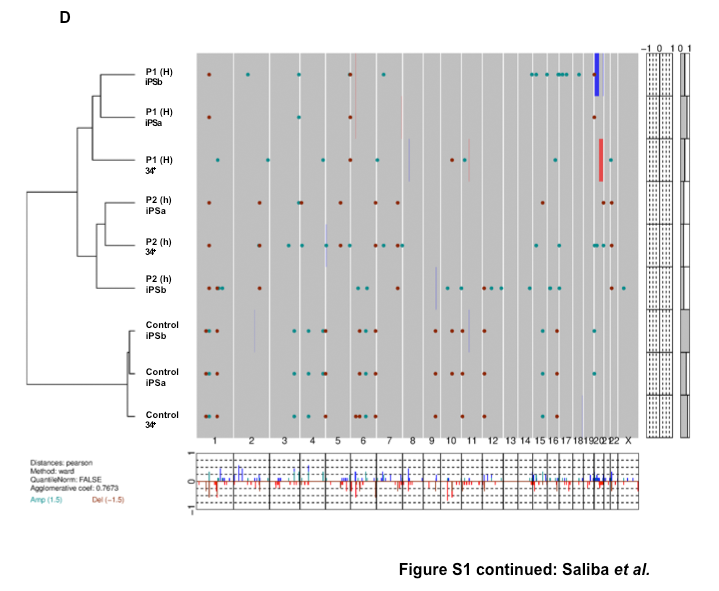

Supplement: Figure S1 — Molecular characterization of iPS. (A) JAK2V617F genotyping of two iPS cells from P1(H), P2(h) or control using a real-time PCR single nucleotide polymorphism detection system. For controls, DNA from a JAK2V617F/V617F mutated sample from the HEL cell line was combined in various proportions with JAK2wt DNA from K562 cells. (B) Karyotypes of iPS from control, P1(H) and P2(h). (C) FISH analysis of iPSb from P1(H) hybridized with subtelomere specific probes for chromosome 20 p-arm (green) and q-arm (red) and BlueFish proximal specific probes RP11-108H13 (red) and RP11-327D19 (green). (D) CGH array analysis is represented by hierarchical Clustering with Pearson distance on 9 samples including two iPS clones and their respective CD34+ progenitors cells from P1(H), P2(h) and control. Dots correspond to amplifications (in green, log2ratio>1.5) and deletions (in red, log 2 r<-1.5) and lines correspond to gains (in blue, 0<log2ratio<1.5) and loss (in red, 0>log2r>-1.5). (DOCX) [file pone.0074257.s001.docx]
